# *trans*-4-[(Phenyl­sulfon­yloxy)meth­yl]cyclo­hexa­necarboxylic acid

**DOI:** 10.1107/S1600536808024513

**Published:** 2008-08-06

**Authors:** Yu-Feng Liang, Qing-Rong Qi, Hu Zheng

**Affiliations:** a Department of Medicinal Chemistry, West China School of Pharmacy, Sichuan University, Chengdu 610041, People’s Republic of China

## Abstract

The title compound, C_14_H_18_O_5_S, is an important inter­mediate for the synthesis of poly(amido­amine) dendrimers. The cyclo­hexane ring adopts a chair conformation with its two substituents in equatorial positions. In the crystal structure, mol­ecules form centrosymmetric dimers via O—H⋯O hydrogen bonds.

## Related literature

For related literature, see: Ahmed *et al.* (2001 ▶); Grabchev *et al.* (2003 ▶); Wang *et al.* (2004 ▶).
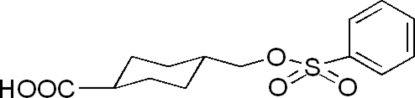


## Experimental

### 

#### Crystal data

C_14_H_18_O_5_S
*M* *_r_* = 298.34
Monoclinic, 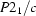

*a* = 17.097 (5) Å
*b* = 5.960 (3) Å
*c* = 14.919 (4) Å
β = 107.09 (3)°
*V* = 1453.2 (10) Å^3^
*Z* = 4
Mo *K*α radiation
μ = 0.24 mm^−1^
*T* = 292 (2) K
0.32 × 0.32 × 0.13 mm

#### Data collection

Enraf–Nonius CAD-4 diffractometer
Absorption correction: none
3702 measured reflections
2691 independent reflections
1323 reflections with *I* > 2σ(*I*)
*R*_int_ = 0.008
3 standard reflections every 250 reflections intensity decay: 0.9%

#### Refinement

*R*[*F* ^2^ > 2σ(*F* ^2^)] = 0.054
*wR*(*F* ^2^) = 0.142
*S* = 1.00
2691 reflections
183 parameters
H-atom parameters constrained
Δρ_max_ = 0.27 e Å^−3^
Δρ_min_ = −0.25 e Å^−3^

### 

Data collection: *DIFRAC* (Gabe & White, 1993 ▶); cell refinement: *DIFRAC*; data reduction: *NRCVAX* (Gabe *et al.*, 1989 ▶); program(s) used to solve structure: *SHELXS97* (Sheldrick, 2008 ▶); program(s) used to refine structure: *SHELXL97* (Sheldrick, 2008 ▶); molecular graphics: *ORTEP-3 for Windows* (Farrugia, 1997 ▶); software used to prepare material for publication: *SHELXL97*.

## Supplementary Material

Crystal structure: contains datablocks global, I. DOI: 10.1107/S1600536808024513/bt2749sup1.cif

Structure factors: contains datablocks I. DOI: 10.1107/S1600536808024513/bt2749Isup2.hkl

Additional supplementary materials:  crystallographic information; 3D view; checkCIF report

## Figures and Tables

**Table 1:** Hydrogen-bond geometry (Å, °)

*D*—H⋯*A*	*D*—H	H⋯*A*	*D*⋯*A*	*D*—H⋯*A*
O4—H4⋯O5^i^	0.82	1.86	2.677 (3)	174

Symmetry code: (i) 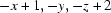.

**Fig. 1.**
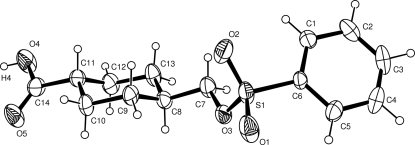
The molecular structure of the title compound with displacement ellipsoids drawn at the 50% probability level.

**Fig. 2.**
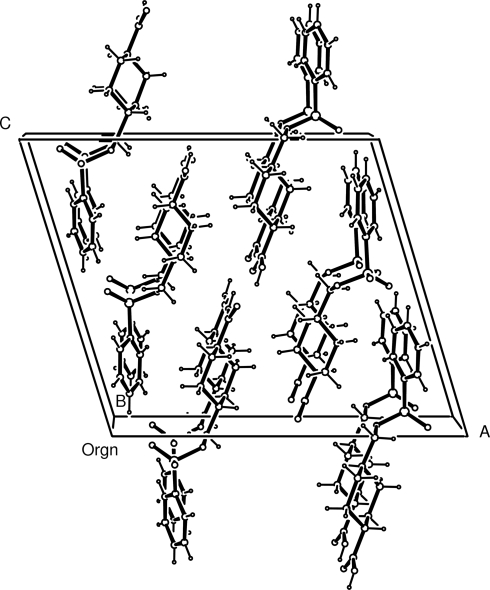
A packing diagram of the title compound. Intermolecular hydrogen bonds are shown as dashed lines.

## supplementary crystallographic information

### Comment

PAMAM (poly(amidoamine)) dendrimers have attracted much interest for their symmetry, high degree of branching and high density of terminal functional groups, which can participate in different reactions. The modification of periphery of PAMAM dendrimer which aimed to change the physical or chemical properties of PAMAM dendrimers, have been reported recently (Grabchev *et al.*,2003; Ahmed *et al.*,2001; Wang *et al.*,2004). To improve the lipophilicity of PAMAM dendrimers and provide a new type of linker with special stereostructure, a series of cyclohexane derivatives were synthesized.

The crystal structure shows that molecules are mainly linked by O—H···.O hydrogen bonds and the cyclohexane ring of the title compound exists in the chair conformation.

### Experimental

*trans*-4-(methoxycarbonyl)cyclohexanemethanol (10 mmol), triethylamine (10 mmol) and a small amount of trimethylamine hydrochloride were suspended in dichloromethane (20 mL), benzenesulfonyl chloride (11 mmol) was dropped with vigorous stirring at room temperature, after 1 h the reaction was quenched by addition of water. The organic layer separated was evaporated to give an oil and the oil was hydrolyzed in methanol and aqueous NaOH (11 mmol) solution for 5 h at 323 K. Then the title compound was obtained by acidification with hydrochloride and recrystallized from acetone. Colorless crystals suitable for X-ray analysis were obtained by slow evaporation in cyclohexane and acetone at room temperature.

### Refinement

H atoms were positioned geometrically (C—H = 0.93–0.98 Å, O—H = 0.82 Å) and refined using a riding model, with *U*_iso_(H) = 1.2*U*_eq_(C,O).

### Crystal data

**Table:**

C_14_H_18_O_5_S	*F*_000_ = 632
*M**_r_* = 298.34	*D*_x_ = 1.364 Mg m^−^^3^
Monoclinic, *P*2_1_/*c*	Mo *K*α radiation λ = 0.71073 Å
*a* = 17.097 (5) Å	Cell parameters from 24 reflections
*b* = 5.960 (3) Å	θ = 4.4–8.7º
*c* = 14.919 (4) Å	µ = 0.24 mm^−^^1^
β = 107.09 (3)º	*T* = 292 (2) K
*V* = 1453.2 (10) Å^3^	Block, colourless
*Z* = 4	0.32 × 0.32 × 0.13 mm

### Data collection

**Table:**

Enraf–Nonius CAD-4 diffractometer	*R*_int_ = 0.008
Radiation source: fine-focus sealed tube	θ_max_ = 25.5º
Monochromator: graphite	θ_min_ = 1.3º
*T* = 292(2) K	*h* = −2→20
ω/2θ scans	*k* = −7→0
Absorption correction: none	*l* = −18→17
3702 measured reflections	3 standard reflections
2691 independent reflections	every 250 reflections
1323 reflections with *I* > 2σ(*I*)	intensity decay: 0.9%

### Refinement

**Table:**

Refinement on *F*^2^	Hydrogen site location: inferred from neighbouring sites
Least-squares matrix: full	H-atom parameters constrained
*R*[*F*^2^ > 2σ(*F*^2^)] = 0.054	*w* = 1/[σ^2^(*F*_o_^2^) + (0.0729*P*)^2^] where *P* = (*F*_o_^2^ + 2*F*_c_^2^)/3
*wR*(*F*^2^) = 0.143	(Δ/σ)_max_ < 0.001
*S* = 1.00	Δρ_max_ = 0.27 e Å^−^^3^
2691 reflections	Δρ_min_ = −0.25 e Å^−^^3^
183 parameters	Extinction correction: SHELXL97 (Sheldrick, 2008), Fc^*^=kFc[1+0.001xFc^2^λ^3^/sin(2θ)]^-1/4^
Primary atom site location: structure-invariant direct methods	Extinction coefficient: 0.0076 (16)
Secondary atom site location: difference Fourier map	

### Special details

**Table:**

Geometry. All e.s.d.'s (except the e.s.d. in the dihedral angle between two l.s. planes) are estimated using the full covariance matrix. The cell e.s.d.'s are taken into account individually in the estimation of e.s.d.'s in distances, angles and torsion angles; correlations between e.s.d.'s in cell parameters are only used when they are defined by crystal symmetry. An approximate (isotropic) treatment of cell e.s.d.'s is used for estimating e.s.d.'s involving l.s. planes.
Refinement. Refinement of *F*^2^ against ALL reflections. The weighted *R*-factor *wR* and goodness of fit *S* are based on *F*^2^, conventional *R*-factors *R* are based on *F*, with *F* set to zero for negative *F*^2^. The threshold expression of *F*^2^ > σ(*F*^2^) is used only for calculating *R*-factors(gt) *etc*. and is not relevant to the choice of reflections for refinement. *R*-factors based on *F*^2^ are statistically about twice as large as those based on *F*, and *R*- factors based on ALL data will be even larger.

### Fractional atomic coordinates and isotropic or equivalent isotropic displacement parameters (Å^2^)

**Table:**

	*x*	*y*	*z*	*U*_iso_*/*U*_eq_	
S1	0.15478 (7)	0.60525 (16)	0.42320 (6)	0.0631 (4)	
O1	0.15724 (19)	0.8419 (4)	0.42806 (17)	0.0942 (11)	
O2	0.10478 (16)	0.4829 (4)	0.46844 (17)	0.0782 (8)	
O3	0.24614 (15)	0.5294 (4)	0.46482 (16)	0.0705 (8)	
O4	0.4357 (2)	−0.1520 (5)	0.8999 (2)	0.1010 (11)	
H4	0.4645	−0.1623	0.9545	0.151*	
O5	0.46270 (19)	0.2062 (5)	0.92678 (18)	0.0973 (11)	
C1	0.0928 (2)	0.3172 (6)	0.2785 (2)	0.0558 (10)	
H1	0.0855	0.2167	0.3231	0.067*	
C2	0.0687 (2)	0.2625 (7)	0.1846 (3)	0.0694 (12)	
H2	0.0457	0.1227	0.1654	0.083*	
C3	0.0785 (3)	0.4130 (9)	0.1199 (3)	0.0786 (14)	
H3	0.0615	0.3752	0.0567	0.094*	
C4	0.1127 (3)	0.6184 (9)	0.1467 (3)	0.0807 (13)	
H4A	0.1184	0.7202	0.1017	0.097*	
C5	0.1386 (2)	0.6739 (6)	0.2393 (2)	0.0636 (11)	
H5	0.1632	0.8122	0.2579	0.076*	
C6	0.12814 (18)	0.5243 (5)	0.3051 (2)	0.0434 (9)	
C7	0.2647 (2)	0.2958 (6)	0.4899 (2)	0.0634 (11)	
H7A	0.2877	0.2257	0.4447	0.076*	
H7B	0.2147	0.2168	0.4885	0.076*	
C8	0.3248 (2)	0.2787 (6)	0.5868 (2)	0.0543 (10)	
H8	0.3740	0.3632	0.5870	0.065*	
C9	0.2910 (2)	0.3764 (6)	0.6619 (2)	0.0560 (10)	
H9A	0.2390	0.3056	0.6579	0.067*	
H9B	0.2812	0.5356	0.6504	0.067*	
C10	0.3488 (2)	0.3433 (6)	0.7602 (2)	0.0595 (11)	
H10A	0.3985	0.4287	0.7669	0.071*	
H10B	0.3232	0.3991	0.8058	0.071*	
C11	0.3702 (2)	0.0971 (6)	0.7794 (2)	0.0540 (10)	
H11	0.3193	0.0165	0.7753	0.065*	
C12	0.4053 (2)	−0.0003 (6)	0.7056 (2)	0.0685 (12)	
H12A	0.4147	−0.1597	0.7169	0.082*	
H12B	0.4577	0.0699	0.7109	0.082*	
C13	0.3484 (2)	0.0350 (6)	0.6063 (2)	0.0688 (12)	
H13A	0.3754	−0.0171	0.5614	0.083*	
H13B	0.2993	−0.0540	0.5981	0.083*	
C14	0.4275 (2)	0.0573 (7)	0.8757 (3)	0.0637 (11)	

### Atomic displacement parameters (Å^2^)

**Table:**

	*U*^11^	*U*^22^	*U*^33^	*U*^12^	*U*^13^	*U*^23^
S1	0.0904 (8)	0.0477 (6)	0.0373 (5)	0.0035 (6)	−0.0028 (5)	−0.0010 (5)
O1	0.152 (3)	0.0478 (16)	0.0584 (17)	0.0040 (17)	−0.0069 (17)	−0.0068 (14)
O2	0.103 (2)	0.0828 (19)	0.0552 (16)	0.0009 (16)	0.0327 (15)	0.0129 (15)
O3	0.0844 (18)	0.0586 (15)	0.0488 (15)	−0.0147 (13)	−0.0111 (13)	0.0161 (12)
O4	0.129 (3)	0.0696 (19)	0.0663 (19)	−0.0009 (17)	−0.0307 (17)	0.0240 (15)
O5	0.125 (3)	0.0689 (18)	0.0601 (18)	−0.0064 (17)	−0.0309 (17)	0.0143 (15)
C1	0.060 (2)	0.053 (2)	0.049 (2)	−0.0035 (18)	0.0074 (18)	0.0019 (18)
C2	0.062 (2)	0.065 (3)	0.065 (3)	0.005 (2)	−0.006 (2)	−0.022 (2)
C3	0.086 (3)	0.104 (4)	0.034 (2)	0.034 (3)	−0.001 (2)	−0.006 (3)
C4	0.105 (3)	0.091 (3)	0.046 (2)	0.020 (3)	0.023 (2)	0.023 (3)
C5	0.077 (3)	0.058 (2)	0.051 (2)	0.004 (2)	0.012 (2)	0.014 (2)
C6	0.049 (2)	0.0432 (19)	0.0327 (18)	0.0033 (16)	0.0031 (15)	0.0025 (15)
C7	0.078 (3)	0.055 (2)	0.046 (2)	0.006 (2)	0.0014 (19)	0.0017 (19)
C8	0.057 (2)	0.053 (2)	0.044 (2)	0.0011 (17)	0.0010 (18)	0.0034 (18)
C9	0.060 (2)	0.054 (2)	0.046 (2)	0.0115 (18)	0.0023 (17)	0.0113 (19)
C10	0.068 (2)	0.064 (2)	0.0378 (19)	0.010 (2)	0.0006 (17)	0.0036 (18)
C11	0.051 (2)	0.057 (2)	0.045 (2)	0.0009 (18)	0.0007 (17)	0.0120 (19)
C12	0.072 (2)	0.063 (2)	0.058 (2)	0.017 (2)	0.000 (2)	0.009 (2)
C13	0.078 (3)	0.067 (3)	0.048 (2)	0.020 (2)	−0.003 (2)	−0.0003 (18)
C14	0.060 (2)	0.066 (3)	0.056 (2)	0.004 (2)	0.0020 (19)	0.020 (2)

### Geometric parameters (Å, °)

**Table:**

S1—O1	1.412 (3)	C7—H7A	0.9700
S1—O2	1.434 (3)	C7—H7B	0.9700
S1—O3	1.568 (3)	C8—C13	1.513 (5)
S1—C6	1.754 (3)	C8—C9	1.521 (5)
O3—C7	1.453 (4)	C8—H8	0.9800
O4—C14	1.295 (4)	C9—C10	1.521 (4)
O4—H4	0.8200	C9—H9A	0.9700
O5—C14	1.209 (4)	C9—H9B	0.9700
C1—C2	1.378 (5)	C10—C11	1.519 (4)
C1—C6	1.380 (4)	C10—H10A	0.9700
C1—H1	0.9300	C10—H10B	0.9700
C2—C3	1.364 (6)	C11—C14	1.501 (4)
C2—H2	0.9300	C11—C12	1.516 (5)
C3—C4	1.365 (6)	C11—H11	0.9800
C3—H3	0.9300	C12—C13	1.529 (4)
C4—C5	1.362 (5)	C12—H12A	0.9700
C4—H4A	0.9300	C12—H12B	0.9700
C5—C6	1.376 (4)	C13—H13A	0.9700
C5—H5	0.9300	C13—H13B	0.9700
C7—C8	1.510 (4)		
			
O1—S1—O2	119.8 (2)	C13—C8—H8	108.2
O1—S1—O3	104.85 (16)	C9—C8—H8	108.2
O2—S1—O3	109.22 (15)	C8—C9—C10	112.4 (3)
O1—S1—C6	108.74 (16)	C8—C9—H9A	109.1
O2—S1—C6	108.57 (16)	C10—C9—H9A	109.1
O3—S1—C6	104.66 (16)	C8—C9—H9B	109.1
C7—O3—S1	119.6 (2)	C10—C9—H9B	109.1
C14—O4—H4	109.5	H9A—C9—H9B	107.9
C2—C1—C6	118.6 (4)	C11—C10—C9	111.1 (3)
C2—C1—H1	120.7	C11—C10—H10A	109.4
C6—C1—H1	120.7	C9—C10—H10A	109.4
C3—C2—C1	120.1 (4)	C11—C10—H10B	109.4
C3—C2—H2	119.9	C9—C10—H10B	109.4
C1—C2—H2	119.9	H10A—C10—H10B	108.0
C2—C3—C4	120.9 (4)	C14—C11—C12	110.4 (3)
C2—C3—H3	119.5	C14—C11—C10	112.7 (3)
C4—C3—H3	119.5	C12—C11—C10	110.9 (3)
C5—C4—C3	119.9 (4)	C14—C11—H11	107.5
C5—C4—H4A	120.1	C12—C11—H11	107.5
C3—C4—H4A	120.1	C10—C11—H11	107.5
C4—C5—C6	119.6 (4)	C11—C12—C13	112.1 (3)
C4—C5—H5	120.2	C11—C12—H12A	109.2
C6—C5—H5	120.2	C13—C12—H12A	109.2
C5—C6—C1	120.8 (3)	C11—C12—H12B	109.2
C5—C6—S1	119.3 (3)	C13—C12—H12B	109.2
C1—C6—S1	119.8 (3)	H12A—C12—H12B	107.9
O3—C7—C8	110.3 (3)	C8—C13—C12	112.1 (3)
O3—C7—H7A	109.6	C8—C13—H13A	109.2
C8—C7—H7A	109.6	C12—C13—H13A	109.2
O3—C7—H7B	109.6	C8—C13—H13B	109.2
C8—C7—H7B	109.6	C12—C13—H13B	109.2
H7A—C7—H7B	108.1	H13A—C13—H13B	107.9
C7—C8—C13	108.5 (3)	O5—C14—O4	122.6 (3)
C7—C8—C9	112.4 (3)	O5—C14—C11	123.5 (3)
C13—C8—C9	111.3 (3)	O4—C14—C11	113.9 (3)
C7—C8—H8	108.2		
			
O1—S1—O3—C7	166.4 (3)	S1—O3—C7—C8	−132.0 (3)
O2—S1—O3—C7	36.9 (3)	O3—C7—C8—C13	−174.7 (3)
C6—S1—O3—C7	−79.2 (3)	O3—C7—C8—C9	61.8 (4)
C6—C1—C2—C3	−1.2 (5)	C7—C8—C9—C10	175.6 (3)
C1—C2—C3—C4	0.6 (6)	C13—C8—C9—C10	53.7 (4)
C2—C3—C4—C5	0.8 (6)	C8—C9—C10—C11	−55.3 (4)
C3—C4—C5—C6	−1.6 (6)	C9—C10—C11—C14	179.8 (3)
C4—C5—C6—C1	1.0 (5)	C9—C10—C11—C12	55.4 (4)
C4—C5—C6—S1	−175.4 (3)	C14—C11—C12—C13	179.5 (3)
C2—C1—C6—C5	0.4 (5)	C10—C11—C12—C13	−54.7 (4)
C2—C1—C6—S1	176.7 (3)	C7—C8—C13—C12	−176.5 (3)
O1—S1—C6—C5	20.1 (3)	C9—C8—C13—C12	−52.4 (4)
O2—S1—C6—C5	151.9 (3)	C11—C12—C13—C8	53.6 (5)
O3—S1—C6—C5	−91.5 (3)	C12—C11—C14—O5	113.9 (5)
O1—S1—C6—C1	−156.3 (3)	C10—C11—C14—O5	−10.8 (6)
O2—S1—C6—C1	−24.4 (3)	C12—C11—C14—O4	−66.6 (5)
O3—S1—C6—C1	92.1 (3)	C10—C11—C14—O4	168.7 (4)

### Hydrogen-bond geometry (Å, °)

**Table:**

*D*—H···*A*	*D*—H	H···*A*	*D*···*A*	*D*—H···*A*
O4—H4···O5^i^	0.82	1.86	2.677 (3)	174

Symmetry codes: (i) −*x*+1, −*y*, −*z*+2.
